# Performance of automated ECV maps versus conventionally calculated ECV

**DOI:** 10.1186/1532-429X-17-S1-P56

**Published:** 2015-02-03

**Authors:** Stefania Rosmini, Thomas A Treibel, Amna Abdel-Gadir, Heerajnarain Bulluck, Daniel Sado, Charlotte Manisty, Anna S Herrey, Peter Kellman, James Moon

**Affiliations:** 1 The Heart Hospital Imaging Centre, University College London Hospitals, London, UK; 2 National Heart, Lung and Blood Institute, Bethesda, MD, USA

## Background

The extracellular volume fraction (ECV) is a surrogate marker for diffuse myocardial fibrosis. ECV is calculated from pre- and post-contrast T1 maps and corrected for hematocrit (Hct). Current workflows are cumbersome, but an automated ECV map tool simplifies this and could promote adoption into routine clinical practice. We assessed the performance of automated ECV maps versus conventional manual ECV quantification in a population of healthy volunteers using a Modified Look-Locker Inversion Recovery (MOLLI) technique.

## Methods

**Figure 1:**
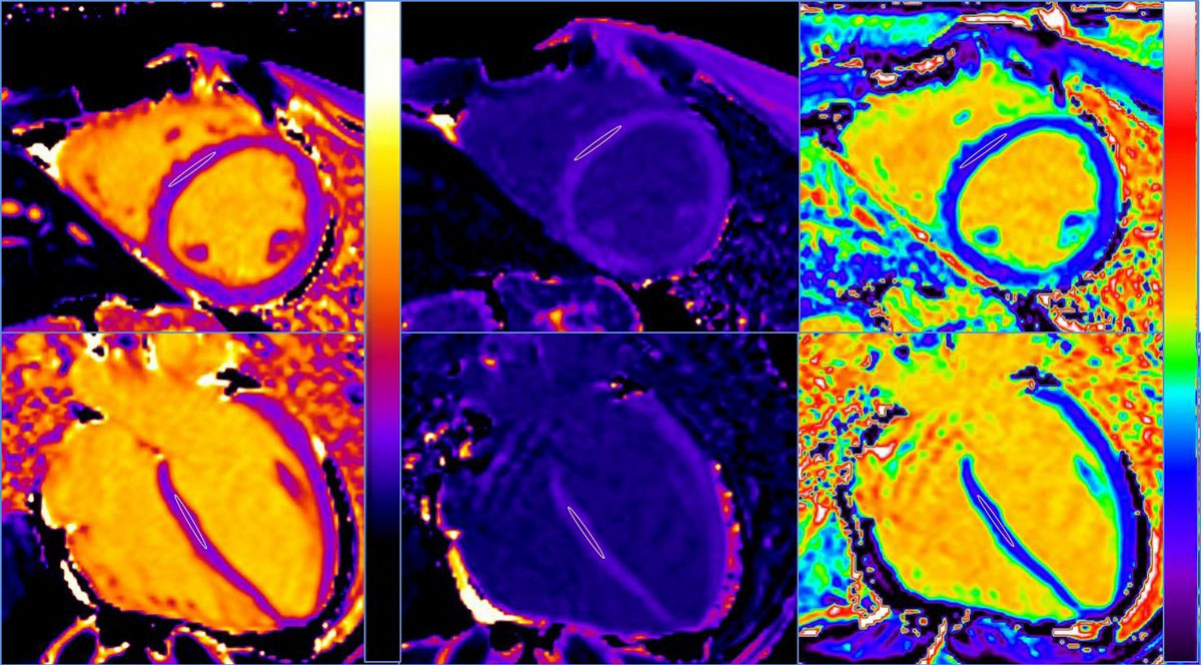
Native T1 maps (left column), post contrast T1 maps (middle column) and ECV maps (right column) in a short axis (top row) and 4 chamber (bottom row).

45 healthy volunteers [mean age 43±12 years, 21 (47%) males] underwent CMR at 1.5T. with T1 mapping using MOLLI. MOLLI T1 maps with motion correction [pre-contrast 5s(3s)3s; post-contrast 4s(1s)3s(1s)2s] were acquired in the 4 chamber (4Ch) and a mid-ventricular short-axis (SA) pre and 15 minutes post contrast (Dotarem 0.1 mmol/kg). A region of interest was manually drawn on the pre-contrast T1 map and a) exported to the post contrast T1 map and b) exported to the ECV map (Figure 1). The ECV map was automatically generated from the source images of the pre and post contrast T1 maps with re-MOCOing and calibrated by blood hematocrit (Kellman JCMR 2012, 14:63). For the manual method, the averages of the ROIs were used in the ECV equation: ECV= (Δ[1/T1_myo_] / Δ[1/T1_blood_]) * [1-hematocrit]). For the ECV map, the ROI pixel average was the average ECV.

## Results

ECV maps resulted in a higher ECV in the SA than in the 4Ch (27.0±3.5% vs 25.9±2.8%; *p*=*0.007*); this difference did not reach significance in the manual technique (27.1±2.8% vs 26.6±2.9%; p=0.075).

**Figure 2:**
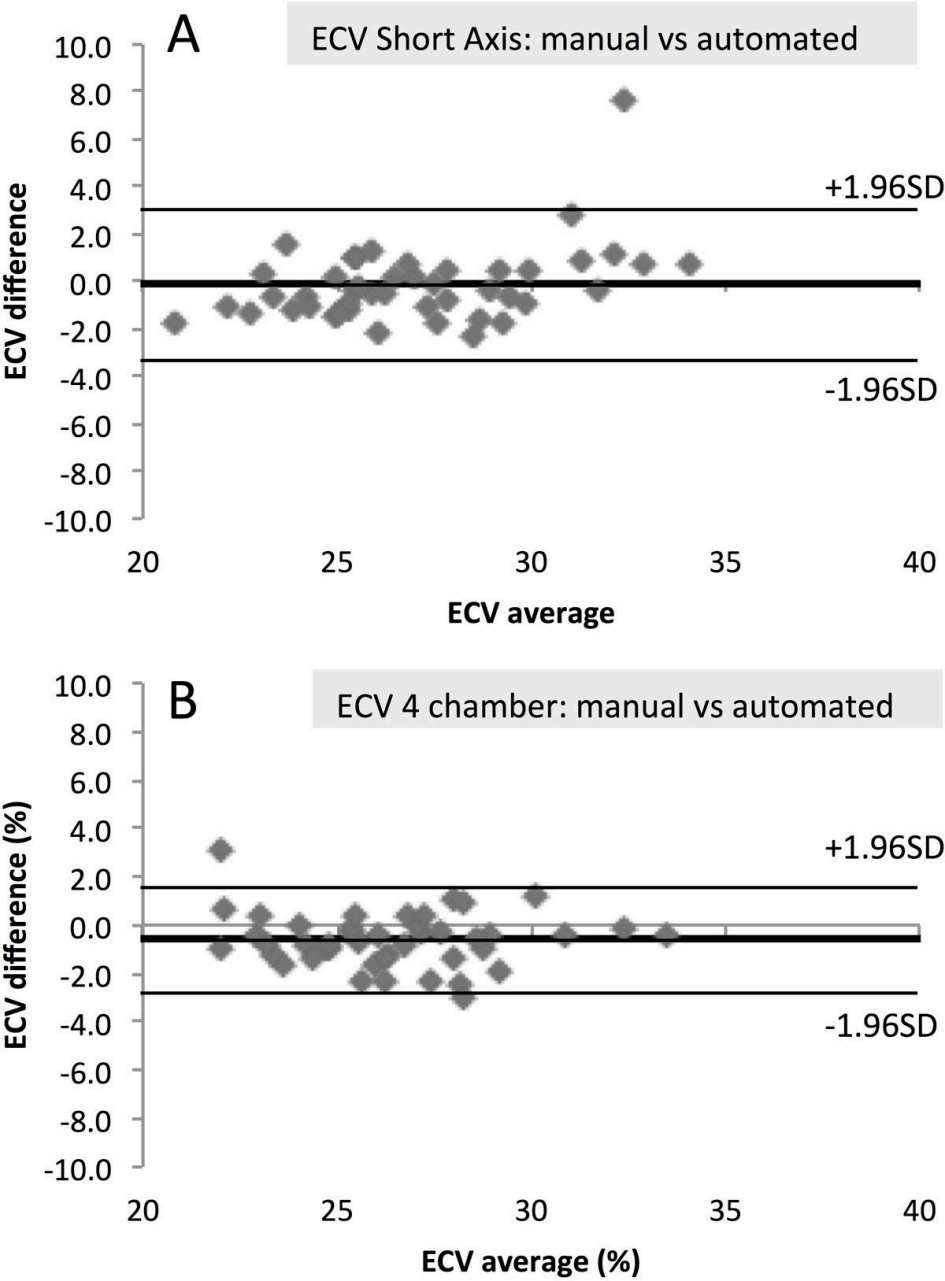
Bland-Altman Plots of ECV by automated vs manual method in the short axis (A) and 4 chamber (B).

In the 4Ch, ECV was higher using the manual rather than the automatic technique (26.6±2.9% vs 25.9±2.8%, *p*<*0.001*). There was excellent correlation between both techniques (R=0.92; p<0.001). Bland Altman analysis revealed minimal bias and no significant variability (bias -0.6%, 95% CI ±2.2%) (Fig. 2B).

In the mid-SA, ECV was not significantly different between manual and automatic technique (27.1±2.8% vs 27.0±3.5%; *p*=*0.546*) with excellent correlation (R= 0.89; p<0.001). Bland Altman analysis revealed no bias or variability (bias -0.15, 95% CI ±3.2%) (Fig. 2A).

## Conclusions

Automated ECV maps significantly improve workflow and show good agreement with the manual method. Application in the short axis showed no difference to the manual approach, but result in slightly higher ECV with more variability than in the 4 chamber. Further validation in health and disease with wider ECV ranges is needed.

## Funding

TAT and MF are supported by doctoral research fellowships by the National Institute of Health Research and British Heart Foundation, respectively.

